# Association of Gestational Hypertension with Sociodemographic and Anthropometric Factors, Perinatal Outcomes, Breastfeeding Practices, and Mediterranean Diet Adherence: A Cross-Sectional Study

**DOI:** 10.3390/medicina59122103

**Published:** 2023-11-30

**Authors:** Eleni Pavlidou, Sousana K. Papadopoulou, Olga Alexatou, Gerasimos Tsourouflis, Georgios Antasouras, Aikaterini Louka, Ioanna P. Chatziprodromidou, Maria Mentzelou, Anastasia Sampani, Maria Chrysafi, Thomas Apostolou, Antonios Dakanalis, Vasiliki G. Papadopoulou, Constantinos Giaginis

**Affiliations:** 1 Department of Food Science and Nutrition, School of the Environment, University of the Aegean, 81400 Myrina, Lemnos, Greece; elen.p.pavl@gmail.com (E.P.); rd.olga.alexatou@gmail.com (O.A.); g.antasouras@gmail.com (G.A.); loukathy612@gmail.com (A.L.); maria.mentzelou@hotmail.com (M.M.); m.chrisafi3@gmail.com (M.C.); 2 Department of Nutritional Sciences and Dietetics, School of Health Sciences, International Hellenic University, 57400 Thessaloniki, Greece; souzpapa@gmail.com (S.K.P.); sousana@the.ihu.gr (V.G.P.); 3 Second Department of Propedeutic Surgery, Medical School, University of Athens, 11527 Athens, Greece; gtsourouflis@med.uoa.gr; 4 Department of Public Health, Medical School, University of Patras, 26504 Patras, Greece; ioannachatzi@med.upatras.gr; 5 First Department of Pathology, Medical School, University of Athens, 11527 Athens, Greece; gerasimos.ts@gmail.com; 6 Department of Physiotherapy, Faculty of Health Sciences, International Hellenic University, 57400 Thessaloniki, Greece; apostolouthomas@hotmail.com; 7 School of Medicine and Surgery, University of Milano-Bicocca, 20900 Monza, Italy; antonios.dakanalis@unimib.it; 8 Fondazione IRCCS San Gerardo dei Tintori, 20900 Monza, Italy

**Keywords:** gestational hypertension, sociodemographic characteristics, anthropometry characteristics, overweight, obesity, gestational weight gain, preterm birth, perinatal outcomes, Mediterranean diet, breastfeeding

## Abstract

*Background and Objectives*: Gestational hypertension has been associated with several pregnancy short-term and long-term complications, affecting both the mother and her infant’s health. The present study aims to assess the potential association of gestational hypertension with sociodemographic and anthropometry factors, perinatal outcomes, breastfeeding habits, and Mediterranean diet (MD) compliance. *Materials and Methods*: This is a cross-sectional study conducted on 5271 mothers that was carried out after delivery. The anthropometry characteristics and perinatal outcomes were retrieved from the mothers’ medical records. Sociodemographic characteristics, MD adherence, and breastfeeding habits were assessed via one-to-one interviews of the assigned women with qualified staff. *Results*: Maternal older age, being employed, family history of gestational hypertension, overweight/obesity before gestation, and abnormal gestational weight gain (GWG) independently increased the risk of developing gestational hypertension. Moreover, gestational hypertension was independently related with a greater incidence of abnormal childbirth body weight and preterm birth, not exclusively breastfeeding, and lower levels of MD adherence. *Conclusions*: This study highlights the importance of informing future mothers about the risk factors of gestational hypertension, underlining also that a healthy lifestyle, which simultaneously includes a healthy nutritional pattern such as MD, may decrease the risk of developing gestational hypertension and the subsequent pregnancy complications.

## 1. Introduction

Hypertensive disorders show a prevalence of 5–10% among all gestations in the world and this incidence seems to continuously be growing worldwide [1,2]. More to the point, hypertensive disorders during gestation consist of gestational hypertension, pre-eclampsia, eclampsia, hemolysis, increased liver enzymes, and reduced platelets syndrome [3]. Among them, gestational hypertension constitutes a common complication of high blood pressure, which can result in serious health risks for both the mother and her baby, being considered an important cause of mother and fetus mortality and morbidity. Remarkably, gestational hypertension seems to be related with a high risk of long-term complications such as cardiovascular disease in women in the later stages of their life [4,5], as well as an increased risk of developing cardiovascular dysfunction in their children [6,7]. Notably, a recent substantial study has found an inverse correlation between cardiovascular health index scores and gestational hypertension, with higher scores associated with lower incidences of gestational hypertension [8]. Alarmingly enough, gestational hypertension may also lead to short-term complications such as placental abruption, hemolysis, increased liver enzymes, decreased platelet count syndrome, preterm birth, intrauterine growth retardation, prematurity, and even fetus or mother mortality [9].

To date, several risk factors have been identified to be associated with high blood pressure during gestation, including maternal pre-pregnancy overweight and obesity, older maternal age, low physical activity levels, systematic smoking, high alcohol consumption, and familial predisposition [10,11,12]. Moreover, several studies have demonstrated that the increasing rates of gestational hypertension have occurred due to changes in certain maternal characteristics and mainly due to the advanced maternal age and the excess pre-pregnancy body weight [13]. In addition, maternal exposure to environmental chemicals such as phthalates, bisphenols, perfluoroalkyl acids, and non-essential metals and trace minerals have been identified as possible risk factors of hypertensive illnesses during gestation [14,15]. There is also evidence that certain genetic risk factors may be implicated in the renin–angiotensin system (RAS), endothelin system, inflammatory factors, and oxidative stress, which could be highly related with gestational hypertension [16,17]. Psychological distress in women during gestation was also shown to act as a possible risk factor for gestational hypertension [18,19]. Additionally, women having a previous history of gestational hypertension have exhibited an increased risk of developing gestational hypertension [20].

Although the etiology of gestational hypertension has not yet been clarified, certain evidence has implied that the maternal nutritional habits may exert a potential role. In this aspect, a substantial systematic review and meta-analysis of observational studies have documented that there are some studies that have supported evidence of the advantageous impact of nutritional habits that include a plethora of plant-based foodstuffs against gestational hypertensive disorders; however, conclusive results cannot be derived yet [21]. In the MoBa survey, nutritional habits that include increased consumption of plant-based foodstuffs and oils have been found to reduce the probability of pre-eclampsia [22]. In contrast, nutritional habits that include an increased intake of processed meat, sweet drinks, and salty snacks may enhance the likelihood of gestational hypertension [22]. In the US cohort study Project Viva, pregnant women with lower age, decreased educational status, who were multiparous, and exhibited increased body mass index (BMI) before gestation were characterized by poorer-quality diets [23]. In the Generation R Study, it was also shown that reduced MD compliance was related with an increased blood pressure during gestation [24]. On the contrary, an increased compliance to a traditional nutritional model, which includes increased consumption of meat and potatoes and reduced consumption of fruits, nonalcoholic drinks, fish, and bread, has been associated with a higher blood pressure during gestation [24].

A case-control study conducted on 100 women with gestational hypertension and 200 normotensive pregnant women was recently performed [25]. This study showed that gestational hypertension was positively associated with higher energy, fat, and protein intake, while a lower risk of gestational hypertension was found for women whose dietary habits included fruits and legumes [25]. In another case-control study, the Dietary Approaches to Stop Hypertension (DASH) diet, an established nutritional pattern, which includes high amounts of whole grains, fruits, vegetables, low-fat dairy products, and plant proteins from nuts and legumes but reduced consumption of red/processed meat, sweets, and sugar-sweetened beverages, was associated with a decreased risk of developing preeclampsia [26].

In general, the etiology and the pathophysiological mechanisms of gestational hypertension have not yet been elucidated, and the specific molecular mechanisms involved in gestational hypertension remain incompletely understood [27,28]. Currently, there are only a few clinical studies evaluating the association of gestational hypertension with sociodemographic and anthropometric factors, perinatal outcomes, breastfeeding practices, and nutritional habits. Moreover, the potential risk factors for developing gestational hypertension have not been well established yet, whereas relevant clinical studies in our country remain extremely scarce. In this aspect, the current cross-sectional survey aims to assess the possible association of gestational hypertension with several sociodemographic and anthropometry factors, and perinatal outcomes. In addition, the present study intends to assess the potential impact of MD against gestational hypertension as well as whether gestational hypertension may affect breastfeeding practices.

## 2. Methods

In the present study, 7348 mothers were initially enrolled from 11 geographically diverse regions of our country, rural, urban and islands (Athens, Thessaloniki, Larissa, Kavala, Alexandroupolis, Ioannina, Patra, Kalamata, Crete, and South and North Aegean). The inclusion criteria for the initial enrollment were women with a singleton birth prior to the assignment, regardless of parity, and without any history of disease except for gestational hypertension. In multiparous women, only the latest gestation was considered. Enrollment to the study ranged between May 2016 and September 2020. All the enrolled women gave birth to a child 2–5 years before the study enrollment.

**Figure 1:**
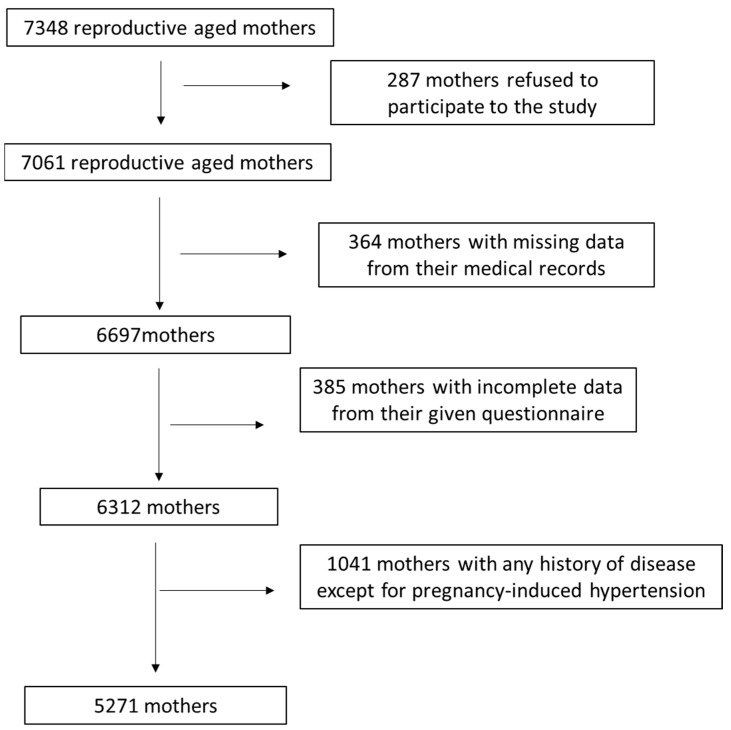
Flow chart diagram of study enrolment.

A thorough description of the survey assignment as a flow chart diagram is represented in Figure 1. By using the appropriate exclusion and inclusion criteria, 5271 mothers were contained in the ending analysis, leading to a final reply rate of 71.7%. The Ethical Agency of the University of the Aegean gave approval for the present survey (ethical approved protocol: no 12/14.5.2016, approval date: 14 May 2016) and was in agreement with the WHO recommendations (52nd WMA General Assembly, Edinburgh, Scotland, 2000).

All the data concerning the assigned women were confidential. All participating mothers had no disease at the time of study, and they were informed about the aim of the survey and signed a consent form. A simple randomization was applied, which is simple and easy to implement in a clinical study. In large clinical studies (n > 200), simple randomization can be trusted to generate adequate numbers of participants among groups. The sample size estimation was carried out utilizing the PS: Power and Sample Size calculator program. The randomization was performed utilizing a sequence of random binary numbers (i.e., 001110110 in which 0 showed assignment and 1 no assignment to the survey). The calculation of the power of our sample size indicated a power of 87.8%.

### 2.1. Study Design

The relevant questionnaires were utilized to gather the sociodemographic characteristics such as age, education status, financial level, ethnicity, marital status, employment status, smoking habits, family history of gestational hypertension, and parity of the assigned women via one-to one interviews of the assigned mothers with the qualified staff to reduce recall biases. The education status was grouped into 3 classes: (a) primary education, (b) secondary education, and (c) university studies. The economic level was classified based on the annual family income as 0 < 5000 €; 1 5000–10,000 €; 2 10,000–15,000 €; 3 15,000–20,000 €; 4 20,000–25,000 €; 5 > 25,000 €. The economic level was additionally grouped as low for family annual income ≤ 10,000 €, medium annual income for 10,000 €–20,000 €, and high annual income for >20,000 €.

The measured body weight and height of the assigned women were derived from their medical records to determine body mass index (BMI) prior to pregnancy. The WHO recommendations were used to categorize the enrolled women as normal weight, overweight, or obese [28,29,30]. Perinatal outcomes such as gestational weight gain (GWG), preterm birth, and mode of delivery (vaginal or caesarean section) were extracted from the women’s medical records. Childbirth weight was also retrieved from the mothers’ medical records and was categorized as low (<2500 g), normal (2500–4000 g), and high (>4000 g), as suggested by the majority of the literature [31].

According to the Institute of Medicine (IOM) recommendations, the recommended GWG for underweight women prior to pregnancy (BMI < 18.5 kg/m^2^) was between 12.5 and 18.0 Kg, for normal weight women (BMI: 18.5–24.9 kg/m^2^) was between 11.6 and 16.0 Kg, for overweight mothers (BMI: 25.0–29.9 kg/m^2^) was in the range of 7.0–11.5 Kg, and for obese women (BMI ≥ 30.0 kg/m^2^) was in the range of 5–9 Kg [32]. The assigned women were categorized based on the aforementioned guidelines into 3 groups: those with decreased GWG than recommended, (b) those inside the recommended range of GWG, and (c) those with higher GWG than recommended.

In addition, the enrolled women answered whether they followed breastfeeding at all and if they have exclusively breastfed their child for at least 4 months [33,34]. To reduce recall biases, the enrolled mothers answered whether they have followed exclusive breastfeeding for their child for at least 4 months. We chose this time point in which the mothers were advised to gradually include pulp foods to the nutritional habits of their children, and thus, they recalled with more validity the lactation period, which has increased the responses’ consistency. In contrast, mothers attempting to breastfeed their infant for lower intervals were not able to answer with adequate consistency regarding the precise interval of breastfeeding [33,34].

The assigned mothers also specified if they had a preterm birth (<37th week) and their answers were further cross-checked with their medical files to establish with more validity the exact week of gestation in which the preterm birth occurred. Nonetheless, there were several missing data concerning the exact week of delivery. In addition, certain medical record data were not in line with the assigned mothers’ responses. Thus, preterm birth was categorized as a binary variable, with birth characterized next to or prior to the 37th week of gestation, which is considered moderate to late preterm birth [33,34].

Regarding MD evaluation, the well-recognized MedDietScore was utilized [35,36]. This questionnaire assesses the food incidence intake of 11 selected food groupings according to the MedDietScore index. Each question includes 6 potential answers, ranking from 0 to 5, dependent on the level of adherence for each food class. The summation of the 11 answers leads to a score from 0 to 55; the higher score reflects elevated MD adherence [35,36]. Regarding cereals, potatoes, fruits, vegetables, dairy, and olive oil, the degrees of 6 possible answers correspond to the daily intake. Concerning legumes, fish, red meat, and poultry, the degrees of 6 possible responses correspond to the weekly intake [35,36]. The 11th question examines wine consumption at a daily incidence with moderate intake (≤1 and ≤2 drinks/day for women and men, respectively; one drink = 100 mL = 12 g ethanol) being considered as the highest score [35,36].

The gestational hypertension data were retrieved from the mothers’ medical records. The blood pressure was determined from the left arm (mmHg) with a mercury sphygmomanometer. Three readings on separate days were performed while each mother was seated near to resting for at least 15 min in relaxed conditions with an empty bladder, and the average of three measurements was finally used [37]. The WHO guidelines were utilized by the qualified personnel for the diagnosis of gestational hypertension [38]. In the current study, gestational hypertension was classified as systolic blood pressure >140 mmHg and diastolic blood pressure >90 mmHg at approximately 20 weeks of pregnancy [39].

The trained staff thoroughly has provided information to all the assigned mothers how to answer the questionnaires in the one-to-one interviews to reduce recall biases. The trained staff has systematically provided detailed information for all the questions concerning every questionnaire to increase the accuracy of responses.

### 2.2. Statistical Analysis

The continuous variables, which met the criteria for normal distribution, were assessed by Student’s *t*-test. The Kolmogorov–Smirnov test was used for evaluating if each continuous variable was normally distributed. The Chi-square test was utilized for categorical variables. The quantitative variables following normal distribution were stated as mean value ± standard deviation (SD). A non-parametric analysis utilizing the Mann–Whitney test was used for the continuous variable, which did not meet the criteria for normal distribution. The quantitative continuous variables that did not follow normal distribution were stated as median value (interquartile range, IQR). The qualitative variables were expressed as absolute or relative frequencies. To assess if gestational hypertension may be associated at an independent level with sociodemographic and anthropometry characteristics, perinatal outcomes, breastfeeding habits, and MD adherence, a multivariate binary logistic regression analysis was used after adjusting for possible confounders. The Statistica 10.0 software, Europe was applied to perform the statistical analysis of all the above data (Informer Technologies, Inc., Hamburg, Germany).

## 3. Results

### 3.1. Descriptive Statistics of the Study Population

This cross-sectional survey conducted on 5271 mothers was accomplished during the postpartum period. Descriptive statistics of the study population are included in Table 1. The mean age of the assigned women was 33.1 ± 5.2 years old (range: 21–48 years old). Most of the assigned women had Greek ethnicity (95.7%), and merely 4.3% of them had another ethnicity. Moreover, 31.5% of the assigned mothers have received primary education, 41.4% of them have completed secondary education, and 27.1% of them have graduated from a university. Additionally, 46.2% of the enrolled women had a decreased financial level, 44.9% of them exhibited a moderate financial level, and merely 8.9% reported an increased financial level. Regarding their marital and employment status, 70.8% of them were married, and 75.8% of them were employed at the time of study. In addition, 74.3% of the assigned mothers were never smokers, whereas 25.7% of them were regular smokers. Furthermore, 69.2% of the enrolled mothers have noted that this was their initial childbirth, and 30.8% have reported they had another previous one or two deliveries. Of the enrolled mothers, 8.4% had a family history of gestational hypertension.

Based on BMI classification before gestation, 72.2% were categorized as normal weight, 18.2% were classified as overweight, and 9.6% were affected by obesity. Based on IOM guidelines, 5.0% of the assigned women exhibited lower GWG than recommended, 66.8% of them exhibited GWG inside the normal range, and 28.2% had higher GWG compared to the recommended. Preterm birth was noted in 18.5% of the assigned women. Moreover, 43.2% of the enrolled women gave birth via caesarean section, and 56.8% of them gave birth via vaginal delivery. Regarding breastfeeding habits for the enrolled women, half of them (50.9%) have followed exclusive breastfeeding for their child for at least 4 months, while 49.1% of them did not follow breastfeeding for their child at all or followed breastfeeding for their child for merely some days or a few weeks. MD adherence did not follow the normal distribution and was categorized into quartiles as (a) very low, (b) low, (c) moderate, and (d) high adherence, and every quartile contained an almost equal number of the assigned women. According to WHO criteria, 8.7% of the enrolled mothers developed gestational hypertension.

### 3.2. Associations of Gestational Hypertension with Sociodemographic Characteristics in the Study Population

Gestational hypertension was considerably more usually observed in older mothers compared to younger mothers (Table 2, *p* = 0.0003). Gestational hypertension was also significantly associated with lower levels of education (Table 2, *p* = 0.0021). Married mothers showed a significantly lower prevalence of gestational hypertension than unmarried mothers (Table 2, *p* = 0.0263). Mothers having a family gestational hypertension history exhibited a considerably higher prevalence of being diagnosed with gestational hypertension (Table 2, *p* < 0.0001). Multiparous women have shown a notably lower prevalence of gestational hypertension compared to nulliparous women (Table 2, *p* = 0.0195). Mothers’ nationality, family economic level, and smoking have not been associated with the incidence of gestational hypertension (Table 2, *p* > 0.05).

### 3.3. Associations of Gestational Hypertension with Anthropometry Characteristics and Perinatal Outcomes in the Study Population

Gestational hypertension was substantially more commonly developed in overweight or obese women pre-pregnancy than normal weight mothers before gestation (Table 2, *p* < 0.0001). In fact, 78.6% of the mothers with gestational hypertension were overweight or obese. In contrast, this prevalence were decreased to 21.4% in mothers who did not develop gestational hypertension (Table 2). Gestational hypertension was also significantly more frequently observed in mothers with lower or excess GWG compared to the mothers with normal GWG (Table 2, *p* < 0.0001). Gestational hypertension was significantly associated with the newborn weight at delivery, as mothers developing gestational hypertension had a significantly higher prevalence of delivering a child with body weight < 2500 g or body weight > 4000 g (Table 2, *p* < 0.0001). Preterm birth was also significantly more frequently observed in mothers with gestational hypertension (Table 2, *p* = 0.0001). Gestational hypertension was not associated with the type of delivery (Table 2, *p* > 0.05). Gestational hypertension was significantly related with a lower prevalence of exclusive breastfeeding for at least 4 months (Table 2, *p* = 0.0009). Finally, gestational hypertension was significantly related with a lower incidence of adopting MD at higher levels (Table 2, *p* < 0.0001).

### 3.4. Multivariate Analysis for Gestational Hypertension after Adjustement for Potential Confounding Factors

In multivariate binary logistic analysis, we have included all the collected parameters for minimizing the potential confounding effects. Gestational hypertension was considerably associated in an independent manner with the women’s age, employment status, family history of gestational hypertension, pre-pregnancy BMI status, GWG, preterm birth, childbirth weight status, breastfeeding habits, and MD adherence (Table 3, *p* < 0.05). On the contrary, gestational hypertension was not independently associated with the women’s nationality, educational and financial level, married status and employment status, parity, smoking, and type of delivery (Table 3, *p* > 0.05).

Older women have exhibited a 31% greater probability of being diagnosed with gestational hypertension compared to younger women (Table 3, *p* = 0021). Employed women exhibited a 27% higher probability of developing gestational hypertension than unemployed women (Table 3, *p* = 0.0247). Mothers having a family history of gestational hypertension showed a more than two-fold risk of developing gestational hypertension (Table 3, *p* = 0.0008). Gestational hypertension was also independently associated with a two-fold higher likelihood of being overweight or obese pre-pregnancy (Table 3, *p* = 0.0085). Moreover, gestational hypertension was considerably related with an 85% greater risk of presenting GWG outside of the IOM recommendations (Table 3, *p* = 0.0105). Gestational hypertension was substantially related with a 75% greater risk of preterm birth (Table 3, *p* = 0.0082). Mothers diagnosed with gestational hypertension have shown a 76% greater probability of delivering a newborn baby with an abnormal body weight (<2500 g or >4000 g) (Table 3, *p* = 0011).

Women with gestational hypertension have shown an approximately two-fold reduced probability of exclusively breastfeeding for at least four months (Table 3, *p* = 0.0086). Moreover, gestational hypertension was substantially related with a more than two-fold reduced likelihood of greater MD compliance (Table 3, *p* = 0.0005).

## 4. Discussion

This is one of the few cross-sectional surveys that examined the possible association of gestational hypertension with several sociodemographic and anthropometric factors, perinatal outcomes, breastfeeding practices, and MD compliance. Maternal older age, being employed, family history of gestational hypertension, pre-pregnancy overweight and obesity, and abnormal GWG were found to independently enhance the probability of being diagnosed with gestational hypertension. Moreover, gestational hypertension was independently related with a greater incidence of abnormal newborn body weight, preterm childbirth, not exclusively breastfeeding, and lower levels of MD adherence. A higher education level, nulliparity, and being married were also associated with low prevalence of gestational hypertension; however, these associations did not remain significant in the multivariate analysis.

In accordance with our study, several studies have supported substantial evidence that advanced maternal age has significantly increased the risk of gestational hypertension [40,41,42,43,44,45]. Notably, gestational hypertension linearly increased for every 5 years age increase concerning pregnant women aged from 30 to 45 years. In agreement with our findings, a ten-fold rise in gestational hypertension risk was noted in women with a BMI ≥ 30 Kg/m^2^ [40]. Accordingly, another study also showed that women’s obesity before gestation was related to a greater probability of gestational hypertension and preeclampsia, which may result in deleterious fetus outcomes [46]. In line with our results, certain studies have shown that mothers having a family history of gestational hypertension or a family background of high blood pressure had a higher risk of developing gestational hypertension [45,47,48,49]. In this aspect, a case-control study showed that women diagnosed with hypertension during gestation may be more probable to develop hypertension in the next stages of their life [50]. Moreover, a cross-sectional study showed a 2.7-fold higher risk of gestational hypertension in pregnant women whose mothers had also developed gestational hypertension [51]. In addition, gestational hypertension was generally considered as an aggravating factor for hypertension, hypercholesterolemia, and diabetes mellitus in the next stages of their life [51].

Accordingly, several other clinical studies verified that overweight and obese women before gestation have a higher probability of developing gestational hypertension [40,46,52,53,54,55,56,57,58,59]. In a case-control survey including 310 pregnant women with gestational hypertension and 930 healthy pregnant women, obese pregnant women were 1.79 times more probable to develop gestational hypertension [60]. Moreover, nulliparous overweight or obese women were 2.13 times more probable to develop gestational hypertension compared to women with normal BMI [60]. In accordance with our study, excess GWG was related with visceral adiposity that could account for 65–75% of the probability of developing gestational hypertension [61]. The potential relevant mechanisms for obesity-associated hypertension may be related to renin–angiotensin–aldosterone system/sympathetic nervous system upregulation, adipokine hyperactivation, insulin tolerance, and immune disturbances, as well as structural and functional kidney, heart, and adipocyte alterations [61,62]. A successful body weight decrease was associated with blood pressure reduction, being considered a multidisciplinary approach, including individualized nutritional interventions, consistent physical activity, and cognitive behavioral treatment [63]. Leptin also appears to exert a critical effect in the enhancement of hypertension in obese pregnancy [64]. A causality impact of genetically predisposed childhood obesity with the probability of hypertension in pregnancy has also been identified [65].

Currently, higher or lower GWG than the IOM recommended levels, is associated with a higher risk of adverse maternal and infant outcomes [66,67]. Characteristically, a systematic review and meta-analysis of more than 1 million pregnant women showed that 47% of them exhibited excessive GWG, while 23% of them exhibited lower GWG compared to IOM references [66]. In a Japanese study, excess GWG increased the risk of early-onset and overall hypertension disorders of pregnancy, while an optimal GWG for reducing pregnancy hypertensive disorders was estimated under 3.85 kg [68]. In addition, above-average GWG was positively correlated with preeclampsia risk [69]. In a case-control survey, the proportion of women having excessive GWG was considerably related with an increased prevalence of gestational hypertension [70]. In a Polish prospective cohort, 36.8% of the enrolled pregnant women had excessive GWG, which increased the risk of gestational hypertension and preeclampsia [46]. In accordance with the above studies, 28.2% of the enrolled mothers showed excessive GWG, which increased the risk of gestational hypertension.

In line with our findings, there is evidence indicating that gestational hypertension increased the risk of preterm birth. In fact, a prospective study including 40 pregnant women with gestational hypertension exhibited an increased risk of preterm birth [71]. On the other hand, in a retrospective study, preterm birth did not show any significant association with gestational hypertension; however, a significant association with the incidence of preeclampsia was noted [72]. In this study, preterm labor was identified as the most common maternal complication, while prematurity was the commonest fetal complication seen in pregnant women with gestational hypertension [72]. In a cross-sectional study including 40,212 deliveries, pregnant women with preeclampsia exhibited a higher risk of preterm birth [42]. Accordingly, a multicenter study showed that gestational hypertension increased the risk of premature birth [41]. Butali et al. also indicated that a prevalence of 16.8% of singleton childbirths were preterm (<37th week of pregnancy), while gestational hypertension was associated with increased odds of preterm birth [43]. Accordingly, another clinical study showed that pregnant women with gestational hypertension exhibited a greater likelihood of preterm delivery compared to normotensive ones [73,74,75].

Our study also found that childbirth body weight was also associated with maternal gestational hypertension, as previously reported by Rahman et al. [76]. In accordance with our findings, a prospective study showed that pregnant women with gestational hypertension exhibited an elevated probability of decreased childbirth weight compared to normotensive ones [74]. In a population-based, case-control study, women giving a child with decreased body weight were 5 times more probable to develop gestational hypertension [75]. A cross-sectional study has also shown that the weight of children born to mothers with gestational hypertension was considerably reduced with an average reduction of 137.45 g [77]. Moreover, in a case-control study, gestational hypertension increased the risk of low childbirth weight [78]. In a cohort study, the mean childbirth weight was also decreased in newborns of mothers with gestational hypertension compared to newborns of normotensive women [79]. In our study, a 50% higher incidence of low and an almost double prevalence of high newborn weight in women with gestational hypertension was found.

In our study, nulliparity was related with an increased prevalence of gestational hypertension. Nevertheless, this association was considerably attenuated after adjusting for several confounding factors. In this aspect, a cross-sectional, prospective study found that gestational hypertension was more common in nulliparous women [71]. Nulliparity was also related with an elevated probability of preeclampsia [56]. However, the currently available data concerning parity remain conflicting, since both nulliparity and multiparity seem to predispose women to a greater probability of preeclampsia [69,80,81]. Accordingly, there are conflicting results concerning the association of gestational hypertension with caesarean section. In a prospective study, women with gestational hypertension showed a higher caesarean delivery rate [79]. Accordingly, two other studies showed that hypertensive mothers had higher rates of caesarean section than normotensive ones [76,82]. However, in our study, we did not find any association or even a trend of correlation between gestational hypertension and caesarean section deliveries.

There is also a questionable issue about smoking habits. Maternal cigarette smoking appeared to decrease the likelihood of gestational hypertension and eclampsia, with a considerable inverse exposure–response relation apparent for preeclampsia or eclampsia [56,83]. In the Boston Birth Cohort, Smoking during gestation was also related with a lower probability of diverse hypertensive disorders, recognized as the “smoking-hypertension paradox” [84]. In the above study, smoking exerted preventive effects against hypertensive diseases among Black participants who used other substances as well, whereas null effects for Hispanic participants were noted [84]. In addition, a US-population-based, retrospective survey indicated that smoking was associated with decreased risk of gestational hypertension [85]. Nevertheless, the above relation was attenuated with elevating childbirth weight centile and did not remain significant at 20th or higher centile among highly systematic smokers, at the 60th or higher centile for moderate and 80th or higher centile for light smokers [85]. A meta-analysis of four cohort studies also suggested the protective effect of smoking longer, smoking more on hypertensive disorders in pregnancy and preeclampsia, repeatedly observed among Europeans and North Americans, an association which was not found in the Japanese [86]. In support of this view, in a Japanese study, women who smoked more than 10 cigarettes per day during gestation exhibited a higher probability of gestational hypertensive disorders [87]. However, a more recent meta-analysis of 13 studies revealed that smoking during pregnancy might prevent gestational hypertensive disorders [88]. Moreover, smoking during gestation was considered a hazard factor for gestational hypertensive disorders in Asia but it was identified as a preventing agent in Europe and North America [88]. However, in the present survey, we did not find any positive or negative association or even a trend of correlation between smoking and gestational hypertension.

There are certain studies highlighting that nutrition may affect the gestational hypertension risk. More to the point, maternal multiple micronutrient supplements and antenatal assessment at the first trimester of gestation seem to exert a protecting effect on both early- and late-onset of gestational hypertension [89]. In a case-control study, saturated fatty acids, such as myristic and palmitic acid, were considerably related with the likelihood of gestational hypertension [90]. In contrast, higher levels of very-long-chain saturated fatty acids, such as arachidic, behenic, and lignoceric acid, were related with a decreased probability of gestational hypertension [90]. Another case-control survey showed that elevated consumption of energy, monounsaturated and polyunsaturated fatty acids, and decreased consumption of vitamin C, potassium, and magnesium were considerably associated with gestational hypertension risk [70]. In an observational, prospective study, consumption of elevated dosages of ω-3 long-chain polyunsaturated fatty acids in the early stages of gestation increased the likelihood of gestational hypertensive disorders [91]. Another prospective, population-based, cohort study demonstrated that elevated consumption of energy, sucrose, and polyunsaturated fatty acids increased the preeclampsia risk [92].

In view of the above consideration, our study found that a higher adherence to MD may decrease the risk of developing gestational hypertension. In this aspect, there is certain substantial evidence supporting that both MD and a dietary approach to stop hypertension (DASH) diet can significantly decrease the risk of hypertension [93]. However, a simple, individualized, Mediterranean-style diet during gestation did not reduce the overall risk of adverse maternal and offspring complications but has the potential to reduce GWG and the risk of gestational diabetes [94]. A recent systematic review and meta-analysis on a nutritional intervention study showed that Mediterranean-style diets and sodium-restriction interventions did not decrease the incidence of gestational hypertension or/and preeclampsia in healthy pregnancies; however, managed nutritional programs reduced the risk of gestational hypertension and preeclampsia [95]. However, the data remain scarce and conflicting so far concerning the impact of MD in the risk of gestational hypertension, and thus, the present study is one of the few studies assessing the MD adherence impact on gestational hypertension risk.

Moreover, the present study is the first study that showed that women presenting with gestational hypertension had a lower probability of exclusively breastfeeding their infant during the lactation period. In this aspect, a recent pilot study found significant differences between milk composition in postpartum women with gestational hypertension compared to normotensive women [96]. Human milk from women with gestational hypertension was found to contain a higher composition of fat, carbohydrates, and energy in comparison to healthy women [96]. Moreover, a prospective study indicated that women with gestational hypertension were at risk of using complementary milk or breastfeeding for shorter durations [97].

The fact should be emphasized that preeclampsia and eclampsia are two of the most serious maternal complications following pregnancy and are the main causes of death in pregnant and delivering women [98]. Although preeclampsia affects 3–8% of pregnancies, the mortality rate of young mothers ranges from 5% to 20% [98]. Thus, identifying the potential risk factors related with sociodemographic, anthropometric, and lifestyle characteristics, including nutritional habits and MD compliance, may exert supplementary beneficial effects in conjunction with the recommended medication treatment against hypertensive disorders during pregnancy.

The current study exhibits specific strengths as it was performed on an adequate sample size of 5271 women assigned from various regions of our country, enhancing the robustness of its findings. The study population was sufficiently representative for our country, including women living in 11 geographically different Greek areas. Another strength of our research is that one-to-one interviews between enrolled mothers and the trained staff were applied for reducing recall biases. The thoroughly explained information and the detailed presentation of the questionnaires that were provided before the one-to-one interviews could additionally minimize potential recall biases, enhancing the validity and the consistency of the mothers’ answers. In addition, our survey contained only healthy women with no history of any serious disorder to minimize comorbidity confounding impacts. The present study has also explored if gestational hypertension may exert an independent impact after adjusting for several possible confounders. In fact, our study considered a wide range of factors, including sociodemographic, anthropometric, and lifestyle-related variables, providing a comprehensive analysis of potential contributors to gestational hypertension. Our study also focused on clinical outcomes such as preterm birth, childbirth weight status, and breastfeeding habits, adding practical relevance to the research and addressing both short-term and long-term implications. Moreover, the emphasis on the MD as a potential protective factor against gestational hypertension aligns with broader public health recommendations for promoting healthy dietary patterns.

Our study has also certain limitations. The cross-sectional design of our study decreased the probability of providing conclusive findings and suffered from potential risks of recall biases, especially regarding the self-reported questions, even if we have applied well-organized one-to-one interviews. More to the point, the reliance on interviews and medical records introduces the possibility of recall bias, particularly for variables dependent on participant recall, such as breastfeeding habits. Nonetheless, self-reported data have comprehensively been used in epidemiological surveys, having adequate reliability and accuracy to estimate diverse outcomes. In addition, the cross-sectional design of our study limits the ability to establish causality. Our study identified associations but cannot definitively determine the cause-and-effect relationships between variables. Another limitation of the present study concerns the fact that BMI was merely used for classifying the assigned women as overweight or obese prior to pregnancy. In this context, body fat mass and distribution could directly be assessed and used in the future studies to expand and confirm our findings. Moreover, the probability remains still of unmeasured confounders such as several aspects of mental health, sleep disturbances, the presence of eating diseases, and the physical activity of the assigned mothers despite our systematic approaches to confounder adjusting. Thus, it is still possible that residual confounding could affect our findings, even if we have applied a thorough adjustment for multiple confounding factors. Moreover, there were certain missing and incomplete data concerning the exact week of delivery and some discrepancies between medical records and participant responses, which may impact the accuracy of our results. Finally, the generalizability of our study may be limited to the specific population studied, impacting its broader applicability.

## 5. Conclusions

This study is one of the few cross-sectional studies and the first study in Greece that has examined the possible association of gestational hypertension with several sociodemographic and anthropometric factors, perinatal outcomes, breastfeeding habits, and MD compliance. Several risk factors for gestational hypertension were identified. In parallel, gestational hypertension was recognized as a risk factor for certain pregnancy complications. It was indicated for the first time that adopting a healthy dietary pattern such as MD may reduce the risk of developing gestational hypertension. In this aspect, public strategies and policies may inform the future mothers about the beneficial effects of MD adherence in combination with other lifestyle factors, e.g., physical activity, against the development of gestational hypertension. Well-organized strategies and policies should further inform the future mothers of the potential risk factors that may lead to gestational hypertension. Moreover, prospective clinical studies should be performed to establish causation between gestational hypertension and the undersigned risk factors.

## Figures and Tables

**Table 1:** Descriptive statistics of the study population.

Characteristics (n = 5271)	Descriptive Statistics
**Age (mean ± SD; years)**	33.1 ± 5.2
**Nationality (n, %)**	
Greek	5042 (95.7%)
Other	229 (4.3%)
**Education level (n, %)**	
Primary education	1662 (31.5%)
Secondary education	2181 (41.4%)
University studies	1428 (27.1%)
**Family economic status (n, %)**	
Low	2436 (46.2%)
Medium	2364 (44.9%)
High	471 (8.9%)
**Marital status (n, %)**	
Married	3734 (70.8%)
Other	1537 (29.2%)
**Employment status (n, %)**	
Employed	3994 (75.8%)
Unemployed	1277 (24.2%)
**Smoking habits (n, %)**	
No smokers	3919 (74.3%)
Smokers	1352 (25.7%)
**Family history of gestational hypertension (n, %)**	
No	4830 (91.6%)
Yes	441 (8.4%)
**Parity (n, %)**	
Nulliparity	3697 (69.2%)
Multiparity	1624 (30.8%)
**Pre-pregnancy BMI status (n, %)**	
Normal weight	3804 (72.2%)
Overweight	962 (18.2%)
Obese	505 (9.6%)
**Gestational weigh gain (n, %)**	
Low	264 (5.0%)
Normal	3519 (66.8%)
Excess	1488 (28.2%)
**Preterm birth (<37th week, n, %)**	
No	4298 (81.5%)
Yes	973 (18.5%)
**Mode of delivery (n, %)**	
Vaginal	2992 (56.8%)
Caesarean section	2279 (43.2%)
**Exclusive breastfeeding (n, %)**	
No	2586 (49.1%)
Yes	2685 (50.9%)
**Childbirth weight status (n, %)**	
Low newborn weight (<2500 g)	439 (8.3%)
Normal newborn weight (2500–4000 g)	4534 (86.0%)
High newborn weight (>4000 g)	298 (5.7%)
**Mediterranean Diet adherence (n, %)**	
Very low	1282 (24.3%)
Low	1319 (25.0%)
Moderate	1353 (25.7%)
High	1317 (25.0%)
**Gestational hypertension (n, %)**	
No	4813 (91.3%)
Yes	458 (8.7%)

**Table 2:** Associations of gestational hypertension with sociodemographic and anthropometry parameters, perinatal outcomes, breastfeeding practices, and MD adherence of the study population.

Characteristics (n = 5271)	Gestational Hypertension		
	No (91.3%)	Yes (8.7%)	*p*-Value
**Age (mean ± SD; years)**	32.8 ± 4.2	33.7 ± 4.5	*p* = 0.0003
**Nationality (n, %)**			*p* = 0.4849
Greek	4601 (95.6%)	441 (96.3%)	
Others	212 (4.4%)	17 (3.7%)	
**Educational level (n, %)**			*p* = 0.0021
Primary education	1498 (31.1%)	164 (35.8%)	
Secondary education	2008 (41.7%)	173 (37.8%)	
University studies	1307 (27.2%)	121 (26.4%)	
**Family economic status (n, %)**			*p* = 0.1347
Low	2209 (45.9%)	227 (49.5%)	
Medium	2164 (45.0%)	200 (43.7%)	
High	440 (9.1%)	31 (6.8%)	
**Marital status (n, %)**			*p* = 0.0263
Married	3458 (71.8%)	276 (60.3%)	
Other	1355 (28.2%)	182 (39.7%)	
**Employment status (n, %)**			*p* = 0.0012
Employed	3603 (74.9%)	391 (85.4%)	
Unemployed	1210 (25.1%)	67 (14.6%)	
**Smoking habits (n, %)**			*p* = 0.3389
Non smokers	3587 (74.5%)	332 (72.5%)	
Smokers	1226 (25.5%)	126 (27.5%)	
**Family history of gestational hypertension (n, %)**			*p* < 0.0001
No	4749 (98.7%)	81 (17.7%)	
Yes	64 (1.3%)	377 (82.3%)	
**Parity (n, %)**			*p* = 0.0195
Nulliparity	3248 (67.5%)	399 (87.1%)	
Multiparity	1565 (32.5%)	59 (12.9%)	
**Pre-pregnancy BMI status (n, %)**			*p* < 0.0001
Normal weight	3706 (77.0%)	98 (21.4%)	
Overweight	818 (17.0%)	144 (31.4%)	
Obese	289 (6.0%)	216 (47.2%)	
**Gestational weigh gain (n, %)**			*p* < 0.0001
Low	208 (4.3%)	56 (12.2%)	
Normal	3258 (67.7%)	261 (57.0%)	
Excess	1347 (28.0%)	141 (30.8%)	
**Preterm birth (<37th week, n, %)**			*p* = 0.0001
No	3969 (82.5%)	329 (71.8%)	
Yes	844 (17.5%)	129 (28.2%)	
**Mode of delivery (n, %)**			*p* = 0.9293
Vaginal	2733 (56.8%)	259 (56.6%)	
Caesarean section	2080 (43.2%)	199 (43.4%)	
**Childbirth weight status (n, %)**			*p* < 0.0001
Low newborn weight (<2500 g)	384 (8.0%)	55 (12.0%)	
Normal newborn weight (2500–4000 g)	4180 (86.8%)	354 (77.3%)	
High newborn weight (>4000 g)	249 (5.2%)	49 (10.7%)	
**Exclusive breastfeeding (n, %)**			*p* = 0.0009
No	2327 (48.3%)	259 (56.6%)	
Yes	2486 (51.7%)	199 (43.4%)	
**Mediterranean Diet compliance (n, %)**			*p* < 0.0001
Very low	1118 (23.2%)	164 (35.8%)	
Low	1147 (23.8%)	172 (37.6%)	
Moderate	1299 (27.0%)	54 (11.8%)	
High	1249 (26.0%)	68 (14.8%)	

**Table 3:** Multivariate logistic regression analysis for assessing whether gestational hypertension may independently be related with sociodemographic and anthropometry parameters, perinatal outcomes, breastfeeding habits, and MD compliance after adjustment for potential confounders.

Characteristics	Gestational Hypertension	
	(No/Yes)	
	RR * (95% CI **)	***p*-Value**
**Age** (Below/Above mean value)	1.31 (1.06–1.59)	*p* = 0.0021
**Ethnicity** (Greek/Other)	0.97 (0.37–1.72)	*p* = 0.6859
**Educational level**	1.12 (0.62–1.67)	*p* = 0.1109
(University Studies/Primary + Secondary Education)		
**Financial level** (High/Low + Medium)	1.21 (0.62–1.85)	*p* = 0.2857
**Marital status** (Married/Other)	1.18 (0.58–1.81)	*p* = 0.3748
**Employment status** (Unemployed/Employed)	1.27 (0.92–1.61)	*p* = 0.0247
**Smoking** (Non smokers/Smokers)	1.05 (0.52–1.63)	*p* = 0.4833
**Family history of gestational hypertension** (No/Yes)	2.21 (1.98–2.45)	*p* = 0.0008
**Parity** (Nulliparity/Multiparity)	0.88 (0.29–1.32)	*p* = 0.1209
**Pre-pregnancy BMI status**	2.08 (1.91–2.27)	*p* = 0.0085
(Normal weight/Overweight + Obese)		
**Gestational weigh gain** (Normal/Low + Excess)	1.85 (1.61–2.06)	*p* = 0.0105
**Preterm birth** (No/Yes)	1.75 (1.48–2.05)	*p* = 0.0082
**Type of delivery** (Vaginal/Caesarean Section)	0.95 (0.29–1.72)	*p* = 0.8747
**Childbirth weight status** (normal/low + High)	1.76 (1.53–1.98)	*p* = 0.0011
**Exclusive breastfeeding** (Yes/No)	2.12 (0.91–2.33)	*p* = 0.0086
**Mediterranean diet compliance**	2.25 (2.06–2.43)	*p* = 0.0005
(Moderate + High/Low + Very Low)		

* Relative Risk: RR. ** CI: Confidence Interval.

## Data Availability

The data presented in this study are available on request from the corresponding author. The data are mot publicly available due to the fact that some of them will be exploited in future studies.

## References

[1] Kintiraki E., Papakatsika S., Kotronis G., Goulis D.G., Kotsis V. (2015). Pregnancy-induced hypertension. Hormones.

[2] Wang W., Xie X., Yuan T., Wang Y., Zhao F., Zhou Z., Hao Zhang H. (2021). Epidemiological trends of maternal hypertensive disorders of pregnancy at the global, regional, and national levels: A population-based study. BMC Pregnancy Childbirth.

[3] Tooher J., Thornton C., Angela Makris A., Ogle R., Korda A., Hennessy A. (2017). All hypertensive disorders of pregnancy increase the risk of future cardiovascular disease. Hypertension.

[4] Behrens I., Basit S., Melbye M., A Lykke J., Wohlfahrt J., Bundgaard H., Thilaganathan B., A Boyd H. (2017). Risk of post-pregnancy hypertension in women with a history of hypertensive disorders of pregnancy: Nationwide cohort study. BMJ.

[5] Lo C.C.W., Lo A.C.Q., Leow S.H., Fisher G., Corker B., Batho O., Morris B., Chowaniec M., Vladutiu C.J., Fraser A. (2020). Future Cardiovascular Disease Risk for Women With Gestational Hypertension: A Systematic Review and Meta-Analysis. J. Am. Heart Assoc..

[6] Benschop L., Schalekamp-Timmermans S., Roeters van Lennep J.E., Jaddoe V.W.V., Steegers E.A.P., Ikram M.K. (2018). Cardiovascular risk factors track from mother to child. J. Am. Heart Assoc..

[7] Miliku K., Bergen N.E., Bakker H., Hofman A., Steegers E.A., Gaillard R., Jaddoe V.W.V. (2016). Associations of maternal and paternal blood pressure patterns and hypertensive disorders during pregnancy with childhood blood pressure. J. Am. Heart Assoc..

[8] Cui K., Huo H., He H., Liu C., Zu C., Wu Y., Zhao H. (2023). Cardiovascular Health Index Scores and Pregnancy-Induced Hypertension: An Association Study. Altern. Ther. Health Med..

[9] American College of Obstetricians and Gynecologists (2020). Gestational Hypertension and Preeclampsia: ACOG Practice Bulletin Summary, Number 222. Obstet. Gynecol..

[10] Dietl A., Farthmann J. (2015). Gestational hypertension and advanced maternal age. Lancet.

[11] Tebeu P.M., Foumane P., Mbu R., Fosso G., Biyaga P.T., Fomulu J.N. (2011). Risk factors for hypertensive disorders in pregnancy: A report from the maroua regional hospital, Cameroon. J. Reprod. Infertil..

[12] Zhuang C., Gao J., Liu J., Wang X., He J., Sun J., Liu X., Liao S. (2019). Risk factors and potential protective factors of pregnancy-induced hypertension in China: A cross-sectional study. J. Clin. Hypertens..

[13] Hutcheon J.A., Lisonkova S., Joseph K.S. (2011). Epidemiology of pre-eclampsia and the other hypertensive disorders of pregnancy. Best Pract. Res. Clin. Obstet. Gynaecol..

[14] Kahn L.G., Trasande L. (2018). Environmental toxicant exposure and hypertensive disorders of pregnancy: Recent findings. Curr. Hypertens. Rep..

[15] Soomro M.H., England-Mason G., Liu J., Reardon A.J., MacDonald A.M., Kinniburgh D.W., Martin J.W., Dewey D. (2023). APrON Study Team Associations between the chemical exposome and pregnancy induced hypertension. Environ. Res..

[16] Yang J., Shang J., Zhang S., Li H., Liu H. (2013). The role of the renin–angiotensin–aldosterone system in preeclampsia: Genetic polymorphisms and microRNA. J. Mol. Endocrinol..

[17] Chen A., Zhao H., Wang J., Zhang R., Liu J., Zhao X., Li C., Jia X., Li X., Lin Y. (2020). Haplotype Analysis of Candidate Genes Involved in Inflammation and Oxidative Stress and the Susceptibility to Preeclampsia. J. Immunol. Res..

[18] Zhang S., Ding Z., Liu H., Chen Z., Wu J., Zhang Y., Yu Y. (2013). Association between mental stress and gestational hypertension/preeclampsia: A meta-analysis. Obstet. Gynecol. Surv..

[19] Pop V.J.M., Boekhorst M.G.B.M., Deneer R., Oei G., Endendijk J.J., Kop W.J. (2022). Psychological Distress During Pregnancy and the Development of Pregnancy-Induced Hypertension: A Prospective Study. Psychosom. Med..

[20] Babore G.O., Aregago T.G., Ermolo T.L., Nunemo M.H., Habebo T.T. (2021). Determinants of pregnancy-induced hypertension on maternal and foetal outcomes in Hossana town administration, Hadiya zone, Southern Ethiopia: Unmatched case-control study. PLoS ONE.

[21] Schoenaker D.A., Soedamah-Muthu S.S., Mishra G.D. (2012). The association between dietary factors and gestational hypertension and pre-eclampsia: A systematic review and meta-analysis of observational studies. BMC Med..

[22] Brantsæter A.L., Haugen M., Samuelsen S.O., Torjusen H., Trogstad L., Alexander J., Magnus P., Meltzer H.M. (2009). A Dietary Pattern Characterized by High Intake of Vegetables, Fruits, and Vegetable Oils Is Associated with Reduced Risk of Preeclampsia in Nulliparous Pregnant Norwegian Women. J. Nutr..

[23] Rifas-Shiman S.L., Rich-Edwards J.W., Kleinman K.P., Oken E., Gillman M.W. (2009). Dietary quality during pregnancy varies by maternal characteristics in Project Viva: A US cohort. J. Am. Diet. Assoc..

[24] Timmermans S., Steegers-Theunissen R.P., Vujkovic M., Bakker R., Breeijen H.D., Raat H., Russcher H., Lindemans J., Hofman A., Jaddoe V.W. (2011). Major dietary patterns and blood pressure patterns during pregnancy: The Generation R Study. Am. J. Obstet. Gynecol..

[25] Nahla K., Ibrahim B.A., Bahaa-Eldin E.A.R. (2022). Association between dietary patterns and pregnancy induced hypertension: A case control study from Sudan. Afr. Health Sci..

[26] Cao Y., Liu Y., Zhao X., Duan D., Dou W., Fu W., Chen H., Bo Y., Qiu Y., Chen G. (2020). Adherence to a Dietary Approaches to Stop Hypertension (DASH)-style Diet in Relation to Preeclampsia: A Case-Control Study. Sci. Rep..

[27] Thakoordeen S., Moodley J., Naicker T. (2018). Candidate gene, genome-wide association and bioinformatic studies in pre-eclampsia: A review. Curr. Hypertens. Rep..

[28] Qu H., Khalil R.A. (2020). Vascular mechanisms and molecular targets in hypertensive pregnancy and preeclampsia. Am. J. Physiol. Heart Circ. Physiol..

[29] World Health Organization (2006). The World Health Report: 2006: Working Together for Health.

[30] James W.P.T. (2008). WHO recognition of the global obesity epidemic. Int. J. Obes..

[31] World Health Organization (1975). International Classification of Disease.

[32] Gilmore L.A., Redman L.M. (2015). Weight gain in pregnancy and application of the 2009 IOM guidelines: Toward a uniform approach. Obesity.

[33] Mantzorou M., Papandreou D., Vasios G.K., Pavlidou E., Antasouras G., Psara E., Taha Z., Poulios E., Giaginis C. (2022). Exclusive Breastfeeding for at Least Four Months Is Associated with a Lower Prevalence of Overweight and Obesity in Mothers and Their Children after 2-5 Years from Delivery. Nutrients.

[34] Papandreou D., Mantzorou M., Tyrovolas S., Pavlidou E., Antasouras G., Psara E., Poulios E., Vasios G.K., Giaginis C. (2022). Pre-Pregnancy Excess Weight Association with Maternal Sociodemographic, Anthropometric and Lifestyle Factors and Maternal Perinatal Outcomes. Nutrients.

[35] Panagiotakos D.B., Pitsavos C., Stefanadis C. (2006). Dietary patterns: A Mediterranean diet score and its relation to clinical and biological markers of cardiovascular disease risk. Nutr. Metab. Cardiovasc. Dis..

[36] Arvaniti F., Panagiotakos D.B. (2008). Healthy indexes in public health practice and research: A review. Crit. Rev. Food Sci. Nutr..

[37] El Bilbeisi A., Hosseini S., Djafarian K. (2017). Association of dietary patterns with diabetes complications among type 2 diabetes patients in gaza strip. Palestine: A cross sectional study. J. Health Popul. Nutr..

[38] DeCherney A.H., Roman A.S., Nathan L., Neri L. (2019). Current Diagnosis & Treatment: Obstetrics & Gynecology.

[39] Sibai B.M. (2003). Diagnosis and management of gestational hypertension and preeclampsia. Obstet. Gynecol..

[40] Bener A., Saleh N.M. (2013). The impact of socio-economic, lifestyle habits, and obesity in developing of pregnancy-induced hypertension in fast-growing country: Global comparisons. Clin. Exp. Obstet. Gynecol..

[41] Spiegler J., Stichtenoth G., Weichert J., König I.R., Schlaud M., Wense A.V., Olbertz D., Gurth H., Schiffmann J.-H., German Neonatal Network, GNN (2013). Pregnancy risk factors for very premature delivery: What role do hypertension, obesity and diabetes play?. Arch. Gynecol. Obstet..

[42] Sutan R., Aminuddin N.A., Mahdy Z.A. (2022). Prevalence, maternal characteristics, and birth outcomes of preeclampsia: A cross-sectional study in a single tertiary healthcare center in greater Kuala Lumpur Malaysia. Front. Public Health.

[43] Butali A., Ezeaka C., Ekhaguere O., Weathers N., Ladd J., Fajolu I., Esezobor C., Makwe C., Odusanya B., Anorlu R. (2016). Characteristics and risk factors of preterm births in a tertiary center in Lagos, Nigeria. Pan Afr. Med. J..

[44] Liu Q., Wang X.X., Zhang Y.K., Li J.H., Wang L. (2018). Correlation between pregnancy-induced hypertension and age in pregnant women from Hebei province, 2016. Zhonghua Liu Xing Bing Xue Za Zhi.

[45] Zeng L., Liao C. (2022). Multivariate logistic regression analysis of preeclampsia in patients with pregnancy induced hypertension and the risk predictive value of monitor-ing platelet, coagulation function and thyroid hormone in pregnant women. Am. J. Transl. Res..

[46] Lewandowska M., Więckowska B., Sajdak S. (2020). Pre-Pregnancy Obesity, Excessive Gestational Weight Gain, and the Risk of Pregnancy-Induced Hypertension and Gestational Diabetes Mellitus. J. Clin. Med..

[47] Gudeta T.A., Regassa T.M. (2019). Pregnancy Induced Hypertension and Associated Factors among Women Attending Delivery Service at Mizan-Tepi University Teaching Hospital, Tepi General Hospital and Gebretsadik Shawo Hospital, Southwest, Ethiopia. Ethiop. J. Health Sci..

[48] Belayhun Y., Kassa Y., Mekonnen N., Binu W., Tenga M., Duko B. (2021). Determinants of Pregnancy-Induced Hypertension among Mothers Attending Public Hospitals in Wolaita Zone, South Ethiopia: Findings from Unmatched Case-Control Study. Int. J. Hypertens..

[49] Grum T., Seifu A., Abay M., Angesom T., Tsegay L. (2017). Determinants of pre-eclampsia/Eclampsia among women attending delivery Services in Selected Public Hospitals of Addis Ababa, Ethiopia: A case control study. BMC Pregnancy Childbirth.

[50] Qasim A., Bashir A., Sajid S., Riaz M.M., Almas A. (2016). Women with pregnancy induced hypertension have a higher risk of developing essential hypertension—A case control study from a tertiary care center in Pakistan. J. Pak. Med. Assoc..

[51] Kurabayashi T., Mizunuma H., Kubota T., Kiyohara Y., Nagai K., Hayashi K. (2013). Pregnancy-induced hypertension is associated with maternal history and a risk of cardio-vascular disease in later life: Japanese cross-sectional study. Maturitas.

[52] Adinegara L.A., Razzak M.S. (2004). Does lifestyle increase the incidence of pregnancy-induced hypertension?. Med. J. Malays..

[53] Liu Y.-H., Liu J.-M., Liu L., Ma R., Ye R.-W., Li S., Chen H., Xue M.-J., Cheng L.-C., Wu L.-M. (2009). The relationship between prepregnancy body mass index and the occurrence of pregnancy induced hypertension. Zhonghua Yu Fang Yi Xue Za Zhi.

[54] Ehrenthal D.B., Jurkovitz C., Hoffman M., Jiang X., Weintraub W.S. (2011). Prepregnancy body mass index as an independent risk factor for pregnancy-induced hypertension. J. Womens Health.

[55] Lucovnik M., Tul N., Verdenik I., Novak Z., Blickstein I. (2012). Risk factors for preeclampsia in twin pregnancies: A population-based matched case-control study. J. Perinat. Med..

[56] Savitz D.A., Danilack V.A., Engel S.M., Elston B., Lipkind H.S. (2013). Descriptive epidemiology of chronic hypertension, gestational hypertension, and preeclampsia in New York State, 1995–2004. Matern. Child Health J..

[57] Kazemian E., Sotoudeh G., Dorosty-Motlagh A.R., Eshraghian M.R., Bagheri M. (2014). Maternal obesity and energy intake as risk factors of pregnancy-induced hypertension among Iranian women. J. Health Popul. Nutr..

[58] Shin D., Song W.O. (2015). Prepregnancy body mass index is an independent risk factor for gestational hypertension, gestational diabetes, preterm labor, and small- and large-for-gestational-age infants. J. Matern. Fetal Neonatal Med..

[59] Shopen N., Schiff E., Koren-Morag N., Grossman E. (2015). Factors That Predict the Development of Hypertension in Women with Pregnancy-Induced Hypertension. Am. J. Hypertens..

[60] Moftakhar L., Solaymani-Dodaran M., Cheraghian B. (2018). Role of obesity in gestational hypertension in primigravidae women: A case control study in Shadegan, Iran. Med. J. Islam. Repub. Iran.

[61] Shams E., Kamalumpundi V., Peterson J., Gismondi R.A., Oigman W., de Gusmão Correia M.L. (2022). Highlights of mechanisms and treatment of obesity-related hypertension. J. Hum. Hypertens..

[62] Becton L.J., Shatat I.F., Flynn J.T. (2012). Hypertension and obesity: Epidemiology, mechanisms and clinical approach. Indian J. Pediatr..

[63] Natsis M., Antza C., Doundoulakis I., Stabouli S., Kotsis V. (2020). Hypertension in Obesity: Novel Insights. Curr. Hypertens. Rev..

[64] Taylor P.D., Samuelsson A.M., Poston L. (2014). Maternal obesity and the developmental programming of hypertension: A role for leptin. Acta Physiol..

[65] Hu B., He X., Li F., Sun Y., Sun J., Feng L. (2023). Childhood obesity and hypertension in pregnancy: A two-sample Mendelian randomization analysis. J. Hypertens..

[66] Goldstein R.F., Abell S.K., Ranasinha S., Misso M., Boyle J.A., Black M.H., Li N., Hu G., Corrado F., Rode L. (2017). Association of Gestational Weight Gain With Maternal and In-fant Outcomes: A Systematic Review and Meta-analysis. JAMA.

[67] Koutelidakis A.E., Alexatou O., Kousaiti S., Gkretsi E., Vasios G., Sampani A., Tolia M., Kiortsis D.N., Giaginis C. (2018). Higher adherence to Mediterranean diet prior to pregnancy is associated with decreased risk for deviation from the maternal recommended gestational weight gain. Int. J. Food Sci. Nutr..

[68] Sugeno M., Kyozuka H., Murata T., Hiraiwa T., Jin T., Fujimori M., Fukumoto Y., Ito F., Suzuki D., Toma F. (2022). Optimal gestational weight gain to reduce the risk of hypertension disorders of pregnancy among women with obesity: A single tertiary referral center study in Japan. J. Obstet. Gynaecol. Res..

[69] Schneider S., Freerksen N., Maul H., Roehrig S., Fischer B., Hoeft B. (2011). Risk groups and maternal-neonatal complications of preeclampsia--current results from the national German Perinatal Quality Registry. J. Perinat. Med..

[70] Kazemian E., Dorosty-Motlagh A.-R., Sotoudeh G., Eshraghian M.-R., Ansary S., Omidian M. (2013). Nutritional status of women with gestational hypertension compared with normal pregnant women. Hypertens. Pregnancy.

[71] Thakur A., Dangal G. (2020). Fetomaternal Outcome in Women with Pregnancy Induced Hypertension versus Normotensive Pregnancy. J. Nepal Health Res. Counc..

[72] Das S., Maharjan R., Bajracharya R., Shrestha R., Karki S., Das R., Odland J.Ø., Odland M.L. (2023). Pregnancy outcomes in women with gestational hypertension and preeclampsia at Paropakar Maternity and Women’s Hospital, Nepal: A retrospective study. PLoS ONE.

[73] Jelliffe-Pawlowski L., Baer R., Blumenfeld Y., Ryckman K., O’Brodovich H., Gould J., Druzin M., El-Sayed Y., Lyell D., Stevenson D. (2015). Maternal characteristics and mid-pregnancy serum biomarkers as risk factors for subtypes of preterm birth. BJOG Int. J. Obstet. Gynaecol..

[74] Berhe A.K., Ilesanmi A.O., Aimakhu C.O., Mulugeta A. (2019). Effect of pregnancy induced hypertension on adverse perinatal outcomes in Tigray regional state, Ethiopia: A prospective cohort study. BMC Pregnancy Childbirth.

[75] El-Qatrawi K.J. (2021). Effect of hypertension on pregnancy outcomes at UNRWA health centres in Gaza governorates: A comparative study. Lancet.

[76] Rahman L.A., Hairi N.N., Salleh N. (2008). Association Between Pregnancy Induced Hypertension and Low Birth Weight; A Population Based Case-Control Study. Asia Pac. J. Public Health.

[77] Lei F., Liu D., Shen Y., Zhang L., Li S., Liu X., Shi G., Li J., Zhao Y., Kang Y. (2018). Study on the Influence of Pregnancy-Induced Hypertension on Neonatal Birth Weight. J. Investig. Med..

[78] El Bilbeisi A.H., Abo Khosa S.M., Taleb M.H., El Afifi A.M. (2023). Assessment of serum, die-tary zinc levels, and other risk factors during the third trimester among pregnant women with and without pregnancy-induced hypertension: A case-control study. Front. Nutr..

[79] Lu C.-Q., Lin J., Yuan L., Zhou J.-G., Liang K., Zhong Q.-H., Huang J.-H., Xu L.-P., Wu H., Zheng Z. (2018). Pregnancy induced hypertension and outcomes in early and moderate preterm infants. Pregnancy Hypertens..

[80] Coghill A.E., Hansen S., Littman A.J. (2011). Risk factors for eclampsia: A population-based study in Washington State, 1987–2007. Am. J. Obstet. Gynecol..

[81] Anderson N.H., Sadler L.C., Stewart A.W., Fyfe E.M., McCowan L.M. (2012). Ethnicity, body mass index and risk of pre-eclampsia in a multiethnic New Zealand population. Aust. N. Z. J. Obstet. Gynaecol..

[82] Ye R.W., Liu Y.H., Ma R., Ren A.G., Liu J.M. (2009). Association between pregnancy-induced hypertension, cesarean delivery and perinatal mortality: A prospective study. Zhong Hua Liu Xing Bing. Xue Za Zhi.

[83] Yang Q., Wen S.W., Smith G.N., Chen Y., Krewski D., Chen X.K., Walker M.C. (2006). Maternal cigarette smoking and the risk of pregnancy-induced hypertension and eclampsia. Int. J. Epidemiol..

[84] Garrison-Desany H.M., Ladd-Acosta C., Hong X., Wang G., Burd I., Sanchez Z.M., Wang X., Surkan P.J. (2023). Addressing the Smoking-Hypertension Paradox in Pregnancy: Insight from a Multiethnic US Birth Cohort. Precis. Nutr..

[85] Peltier M.R., Ananth C.V. (2007). Is the association of maternal smoking and pregnancy-induced hypertension dependent on fetal growth?. Am. J. Obstet. Gynecol..

[86] Morisaki N., Obara T., Piedvache A., Kobayashi S., Miyashita C., Nishimura T., Ishikuro M., Sata F., Horikawa R., Mori C. (2023). Association Between Smoking and Hypertension in Pregnancy Among Japanese Women: A Meta-analysis of Birth Cohort Studies in the Japan Birth Cohort Consortium (JBiCC) and JECS. J. Epidemiol..

[87] Tanaka K., Nishigori H., Watanabe Z., Iwama N., Satoh M., Murakami T., Hamada H., Hoshiai T., Saito M., Japan Environment & Children’s Study Group (2019). Higher prevalence of hypertensive disorders of pregnancy in women who smoke: The Japan environment and children’s study. Hypertens. Res..

[88] Wang J., Yang W., Xiao W., Cao S. (2022). The association between smoking during pregnancy and hypertensive disorders of pregnancy: A systematic review and meta-analysis. Int. J. Gynaecol. Obstet..

[89] Liu Y., Li N., Mei Z., Li Z., Ye R., Zhang L., Li H., Zhang Y., Liu J.M., Serdula M.K. (2021). Effects of prenatal micronutrients supplementation timing on pregnancy-induced hypertension: Secondary analysis of a double-blind randomized controlled trial. Matern. Child Nutr..

[90] Li X., Huang Y., Zhang W., Yang C., Su W., Wu Y., Chen X., Zhou A., Huo X., Xia W. (2020). Association of circulating saturated fatty acids with the risk of pregnancy-induced hypertension: A nested case–control study. Hypertens. Res..

[91] Olafsdottir A., Skuladottir G., Thorsdottir I., Hauksson A., Thorgeirsdottir H., Steingrimsdottir L. (2006). Relationship between high consumption of marine fatty acids in early pregnancy and hypertensive disorders in pregnancy. BJOG Int. J. Obstet. Gynaecol..

[92] Clausen T., Slott M., Solvoll K., Drevon C.A., Vollset S.E., Henriksen T. (2001). High intake of energy, sucrose, and polyunsaturated fatty acids is associated with increased risk of preeclampsia. Am. J. Obstet. Gynecol..

[93] Panbehkar-Jouybari M., Mollahosseini M., Salehi-Abargouei A., Fallahzadeh H., Mirzaei M., Hosseinzadeh M. (2021). The Mediterranean diet and dietary approach to stop hypertension (DASH)-style diet are differently associated with lipid profile in a large sample of Iranian adults: A cross-sectional study of Shahedieh cohort. BMC Endocr. Disord..

[94] Al Wattar B.H., Dodds J., Placzek A., Beresford L., Spyreli E., Moore A., Carreras F.J.G., Austin F., Murugesu N., Roseboom T.J. (2019). Mediterranean-style diet in pregnant women with metabolic risk factors (ESTEEM): A pragmatic multicentre randomised trial. PLoS Med..

[95] Imanpour V., Khoshhali M., Goodarzi-Khoigani M., Kelishadi R. (2023). Systematic review and meta-analysis of nutritional interventions to prevent of gestational hypertension or/and preeclampsia among healthy pregnant women. J. Res. Med. Sci..

[96] Sokołowska E.M., Jassem-Bobowicz J.M., Drążkowska I., Świąder Z., Domżalska-Popadiuk I. (2023). Gestational Hypertension and Human Breast Milk Composition in Correlation with the Assessment of Fetal Growth—A Pilot Study. Nutrients.

[97] Strapasson M.R., Ferreira C.F., Ramos J.G.L. (2018). Feeding practices in the first 6 months after delivery: Effects of gestational hypertension. Pregnancy Hypertens..

[98] Laskowska M. (2023). Eclampsia: A Critical Pregnancy Complication Demanding Enhanced Maternal Care: A Review. Med. Sci. Monit..

